# Supplementary material for: Determinants of mosaic chromosomal alteration fitness
Source: Nat Commun. 2024 May 7;15:3800. doi: 10.1038/s41467-024-48190-8 (PMC11076528; doi:10.1038/s41467-024-48190-8)
Supplement: Supplementary file 3 — Reporting Summary [file 41467_2024_48190_MOESM3_ESM.pdf]

nature portfolio

Corresponding author(s): Alexander Bick

Last updated by author(s): Aug 31, 2023

Reporting Summary

Nature Portfolio wishes to improve the reproducibility of the work that we publish. This form provides structure for consistency and transparency in reporting. For further information on Nature Portfolio policies, see our Editorial Policies and the Editorial Policy Checklist.

Statistics

For all statistical analyses, confirm that the following items are present in the figure legend, table legend, main text, or Methods section.

n/a	Confirmed	
☐	☒	The exact sample size (*n*) for each experimental group/condition, given as a discrete number and unit of measurement
☐	☒	A statement on whether measurements were taken from distinct samples or whether the same sample was measured repeatedly
☐	☒	The statistical test(s) used AND whether they are one- or two-sided<br>*Only common tests should be described solely by name; describe more complex techniques in the Methods section.*
☐	☒	A description of all covariates tested
☐	☒	A description of any assumptions or corrections, such as tests of normality and adjustment for multiple comparisons
☐	☒	A full description of the statistical parameters including central tendency (e.g. means) or other basic estimates (e.g. regression coefficient) AND variation (e.g. standard deviation) or associated estimates of uncertainty (e.g. confidence intervals)
☐	☒	For null hypothesis testing, the test statistic (e.g. *F*, *t*, *r*) with confidence intervals, effect sizes, degrees of freedom and *P* value noted<br>*Give P values as exact values whenever suitable.*
☐	☒	For Bayesian analysis, information on the choice of priors and Markov chain Monte Carlo settings
☐	☒	For hierarchical and complex designs, identification of the appropriate level for tests and full reporting of outcomes
☐	☒	Estimates of effect sizes (e.g. Cohen's *d*, Pearson's *r*), indicating how they were calculated

Our web collection on statistics for biologists contains articles on many of the points above.

Software and code

Policy information about availability of computer code

Data collection	No software was used.
Data analysis	R 4.1.2 and Python 2.7.17 were used for analysis. In addition, several other open source packages were used, linked here: Mutect2 v4.1.8: https://dockstore.org/workflows/github.com/broadinstitute/gatk/mutect2:4.1.8.1?tab=info; R packages used for analysis: ggplot2, data.table, tidyverse, dplyr; Python packages: Pandas, matplotlib, sklearn, scipy, seaborn<br><br>The code to call mosaic chromosomal alterations with MoChA version 1.11 is available here: https://github.com/freeseek/mocha. Passenger count variant calling pipeline is available here: https://github.com/weinstockj/passenger_count_variant_calling. Code for the analyses of this paper can be found here: https://github.com/bicklab/pacer-mca-fitness.

For manuscripts utilizing custom algorithms or software that are central to the research but not yet described in published literature, software must be made available to editors and reviewers. We strongly encourage code deposition in a community repository (e.g. GitHub). See the Nature Portfolio guidelines for submitting code & software for further information.

Data

Policy information about availability of data

All manuscripts must include a data availability statement. This statement should provide the following information, where applicable:

- Accession codes, unique identifiers, or web links for publicly available datasets
- A description of any restrictions on data availability
- For clinical datasets or third party data, please ensure that the statement adheres to our policy

Data for each participating study can be accessed through dbGaP with the corresponding TOPMed accession numbers: Amish (phs000956), ARIC (phs001211), BioMe (phs001644), BAGS (phs001143), CARDIA (phs001612), CFS (phs000954), CHS (phs001368), COPDGene (phs000951), FHS (phs000974), GeneSTAR (phs001218), GENOA (phs001345), GOLDN (phs001359), HCHS/SOL (phs001395), HyperGEN (phs001293), JHS (phs000964), MESA (phs001416), VU_AF (phs001032), WGHS (phs001040) and WHI (phs001237). Controlled-access release to the general scientific community via dbGaP is ongoing. GWAS summary statistics for PACER amongst individuals with mCAs are available in the following GitHub repository here: https://github.com/bicklab/pacer-mca-fitness/blob/main/Data/gwas_results_pacerint_nochip_df.assoc.

Research involving human participants, their data, or biological material

Policy information about studies with human participants or human data. See also policy information about sex, gender (identity/presentation), and sexual orientation and race, ethnicity and racism.

Reporting on sex and gender	Sex was considered as a covariate in all analyses in this study.
Reporting on race, ethnicity, or other socially relevant groupings	Self-reported race was not used in this study, but the first 10 principal components of ancestry were used as covariates in the analyses.
Population characteristics	In TOPMed, the median age was 55 years, the mean age was 52.5 years, and the maximum age was 98. The samples have diverse reported ethnicity (40% European, 32% African, 16% Hispanic/Latino, 10% Asian). The median age of individuals with autosomal mCAs was 67, and 57% were female
Recruitment	In TOPMed, 49 cohorts were included, each with differing sample recruitment protocols. More information can be found at the dbGaP page for each of the constituent cohorts.
Ethics oversight	Each of the 49 cohorts obtained IRB permission from their respective institutions and are supported by the NHLBI. Each of the studies obtained informed consent from each participant. The study design was approved by the Vanderbilt Institutional Review Board (IRB#210270)

Note that full information on the approval of the study protocol must also be provided in the manuscript.

Field-specific reporting

Please select the one below that is the best fit for your research. If you are not sure, read the appropriate sections before making your selection.

☒ Life sciences ☐ Behavioural & social sciences ☐ Ecological, evolutionary & environmental sciences

For a reference copy of the document with all sections, see nature.com/documents/nr-reporting-summary-flat.pdf

Life sciences study design

All studies must disclose on these points even when the disclosure is negative.

Sample size	6,930 participants with mCAs were included based on convenience sampling.
Data exclusions	No data was excluded.
Replication	The experimental data had sufficiently high n to perform statistical comparisons. The genome-wide association studies were performed with sex-stratification in the review process and these attempts were successful.
Randomization	Sample recruitment included no randomization mechanism, but relevant covariates are controlled for in all statistical analyses including genetic sex, ancestry, and age.
Blinding	Investigators were blinded with respect to generation of whole genome sequencing data from the TOPMed participants.

Reporting for specific materials, systems and methods

We require information from authors about some types of materials, experimental systems and methods used in many studies. Here, indicate whether each material, system or method listed is relevant to your study. If you are not sure if a list item applies to your research, read the appropriate section before selecting a response.

Materials & experimental systems

n/a	Involved in the study
☒	☐ Antibodies
☒	☐ Eukaryotic cell lines
☒	☐ Palaeontology and archaeology
☒	☐ Animals and other organisms
☒	☐ Clinical data
☒	☐ Dual use research of concern
☒	☐ Plants

Methods

n/a	Involved in the study
☒	☐ ChIP-seq
☒	☐ Flow cytometry
☒	☐ MRI-based neuroimaging
